# ImageTBAD: A 3D Computed Tomography Angiography Image Dataset for Automatic Segmentation of Type-B Aortic Dissection

**DOI:** 10.3389/fphys.2021.732711

**Published:** 2021-09-27

**Authors:** Zeyang Yao, Wen Xie, Jiawei Zhang, Yuhao Dong, Hailong Qiu, Haiyun Yuan, Qianjun Jia, Tianchen Wang, Yiyi Shi, Jian Zhuang, Lifeng Que, Xiaowei Xu, Meiping Huang

**Affiliations:** ^1^School of Medicine, South China University of Technology, Guangzhou, China; ^2^Guangdong Provincial Key Laboratory of South China Structural Heart Disease, Guangdong Cardiovascular Institute, Guangdong Provincial People's Hospital, Guangdong Academy of Medical Sciences, Guangzhou, China; ^3^School of Computer Science, Fudan University, Shanghai, China; ^4^Department of Computer Science and Engineering, University of Notre Dame, Notre Dame, IN, United States; ^5^Medical Imaging Center, Shenzhen Hospital, Southern Medical University, Shenzhen, China

**Keywords:** type-B aortic dissection, automatic segmentation, computed tomography, dataset, deep neural networks

## Abstract

Type-B Aortic Dissection (TBAD) is one of the most serious cardiovascular events characterized by a growing yearly incidence, and the severity of disease prognosis. Currently, computed tomography angiography (CTA) has been widely adopted for the diagnosis and prognosis of TBAD. Accurate segmentation of true lumen (TL), false lumen (FL), and false lumen thrombus (FLT) in CTA are crucial for the precise quantification of anatomical features. However, existing works only focus on only TL and FL without considering FLT. In this paper, we propose ImageTBAD, the first 3D computed tomography angiography (CTA) image dataset of TBAD with annotation of TL, FL, and FLT. The proposed dataset contains 100 TBAD CTA images, which is of decent size compared with existing medical imaging datasets. As FLT can appear almost anywhere along the aorta with irregular shapes, segmentation of FLT presents a wide class of segmentation problems where targets exist in a variety of positions with irregular shapes. We further propose a baseline method for automatic segmentation of TBAD. Results show that the baseline method can achieve comparable results with existing works on aorta and TL segmentation. However, the segmentation accuracy of FLT is only 52%, which leaves large room for improvement and also shows the challenge of our dataset. To facilitate further research on this challenging problem, our dataset and codes are released to the public (Dataset, 2020).

## 1. Introduction

**Figure 1:**
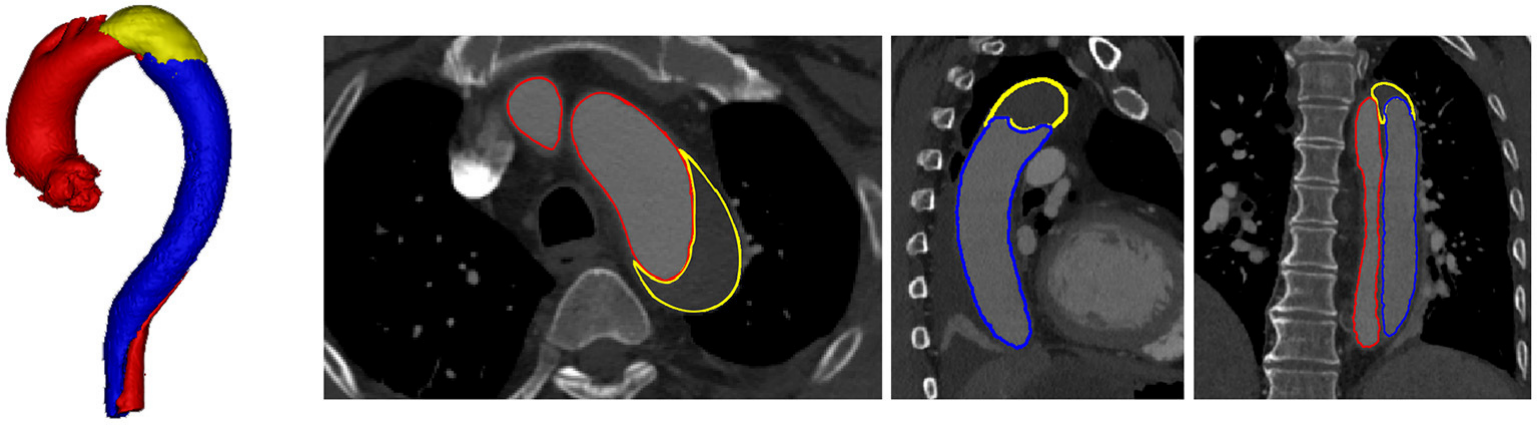
Visualization of TBAD in a 3D model including FLT (yellow), TL (red), and FL (blue), and the corresponding CTA image with axial, coronal, and sagittal views.

Type-B aortic dissection (TBAD) is the surging of blood through a tear in the aortic intima with separation of the intima and media, and creation of a false lumen (channel) as shown in Figure 1, which is one of the most serious cardiovascular events. TBAD affecting 3–4 per 100,000 people per year (Karthikesalingam et al., 2010). Approximately 20% of patients with TBAD die before admission (Karthikesalingam et al., 2010), without treatment, 1–3% patients die per hour during the first 24 h, 30% at the first week, 80% at 2 weeks, and 90% at the first year (Hagan et al., 2000). With the thoracic endovascular aortic repair (TEVAR) surgery and proper treatment patients are reportedly yielding an impressively low 30-day mortality rate of 10% or less (Hagan et al., 2000). Recently, TBAD has attracted a lot of attention due to its growing yearly incidence (Suzuki et al., 2003), and the severity of disease prognosis.

Computed Tomography Angiography (CTA) is routinely adopted for the diagnosis, surgical planning, and prognosis of TBAD. Particularly, quantification assessment of anatomical features in CTA plays a key role in surgical procedure and treatment planning for prognosis. And segmentation of true lumen (TL), false lumen (FL) , and false lumen thrombus (FLT) is a significant step of the quantification assessment. However, manual segmentation by slice is time-consuming and requires expertise, while current computer-aided approaches focus on the segmentation of the entire aorta, which is unable to segment TL, FL, or FLT, automatic segmentation of substructures of TBAD is urgently needed. And there are already some studies trying to solve this problem. Specifically, Melito et al. use the adaptive algorithm together and the meta-model technique of Polynomial-Chaos Kriging define the areas in the cross-section plane in which a point can be used to enrich the dissected segmentation for aorta dissection reconstruction. During establishing the mathematical and computational models of aorta dissection, the level of uncertainty is extremely high. They point out that “One of the leading causes of this uncertainty is the lack of useful datasets” (Melito and Ellermann, 2019). Gamechi et al. propose a fully automatic method combining multi-atlas registration, aorta centerline extraction, and an optimal surface segmentation approach to extract the aorta surface around the centerline. The fully automatic method they propose can assess diameters in the thoracic aorta reliably even in non-ECG-gated, non-contrast CT scans, which could be a promising tool to assess aorta dilatation in screening and in clinical practice. However, the method they propose still has no FLT detection ability mainly due to the lack of FLT enabled dataset (Gamechi et al., 2019). Particularly, there are already some works using neural networks to automatically segment TL, FL, and Aorta (Li et al., 2018; Cao et al., 2019). Li et al. report a fully automatic approach based on a 3-D multi-task deep convolutional neural network that segments the entire aorta and true-false lumen from CTA images in a unified framework. The approach they report achieves a mean dice similarity score(DSC) of 0.910, 0.849, and 0.821 for the entire aorta, true lumen, and false lumen, respectively. Cao et al. also use a convolutional neural network to solve the problems and achieves above 90% of the mean Dice coefficients of each lumen of TBAD when not considering FLT. They provide a promising approach for accurate and efficient segmentation of TBAD and make it possible for automated measurements of TBAD anatomical features. However, existing works nowadays only focus on one of or both TL and FL (Li et al., 2018; Cao et al., 2019; Gamechi et al., 2019; Melito and Ellermann, 2019), and FLT information is poorly explored, partially because of the lack of a dataset. There are some other works considering thrombus in other diseases such as an abdominal aortic aneurysm (Lisowska et al., 2017; Yong et al., 2017; López-Linares et al., 2018), however, TBAD research has not yet advanced to the quantitative measurement of FLT like abdominal aortic aneurysm.

In fact, quantification assessment of FLT is also critical for surgical planning and prognosis. First, the FLT description in clinical radiology reports plays a pivotal role in guiding the endovascular intervention surgery (Dohle et al., 2017). Second, FLT greatly affects patients' postoperative complications (Higashigaito et al., 2019) thus is also a significant independent predictor of post-discharge mortality in prognosis (Trimarchi et al., 2013; Higashigaito et al., 2019). Automatic, efficient, and accurate assessment of FLT is particularly useful for doctors to make a decision on TBAD.

In this paper, we propose ImageTBAD, the first 3D CTA image dataset of TBAD with annotation of TL, FL , and FLT. For simplification of discussion, FL is the part of traditional FL without FLT in our paper. The proposed dataset contains 100 TBAD CTA images, which is of decent size compared with existing medical imaging datasets. Compared with TL and FL, FLT can appear in almost anywhere along the aorta with irregular shapes, which introduces many challenges to accurate segmentation of it. FLT segmentation represents a wide class of segmentation problems where targets exist in a variety of positions with irregular shapes. We further proposed a baseline method based on 3D U-net (Çiçek et al., 2016) for automatic segmentation of TBAD. Results show that the baseline method can achieve comparable results with existing works on the aorta and TL segmentation. However, the segmentation accuracy of FLT is the only 52%, which leaves large room for improvement and also shows the challenge of our dataset. To facilitate further research on this challenging topic, our dataset and codes are released to the public (Dataset, 2020).

## 2. The Image-TBAD Dataset

The ImageTBAD dataset consists of a total of 100 3D CTA images gathered from Guangdong Provincial Peoples' Hospital from January 1, 2013, to April 23, 2015. Images are acquired from two kinds of scanners (Siemens SOMATOM Force, and Philips 256-slice Brilliance iCT system), the characteristics of the ImageTBAD dataset is detailed in Table 1. All the images are pre-operative TBAD CTA images whose top and bottom are around the neck and the brachiocephalic vessels, respectively, in the axial view. The segmentation labeling is performed by a team of two cardiovascular radiologists who have extensive experience with TBAD. The segmentation label of each image is fulfilled by one radiologist and checked by the other. The time to label each image is around 1–1.5 h. The segmentation includes three substructures: TL, FL, and FLT. There are 68 images containing FLT while 32 images are free of FLT.

**Table 1:** Characteristics of the ImageTBAD dataset.

**Parameters**	***N***
Sex = Female (%)	31 (31%)
Age (*Mean ± SD*)	52.5 ± 11.3
Manufacturer = Philips (%)	77 (77%)
Spacing between slice (*mm*)	0.75
Size of the images (*pixels*)	512 × 512 × (135–416)
Typical voxel size (*mm*^3^)	0.25 × 0.25 × 0.25

**Figure 2:**
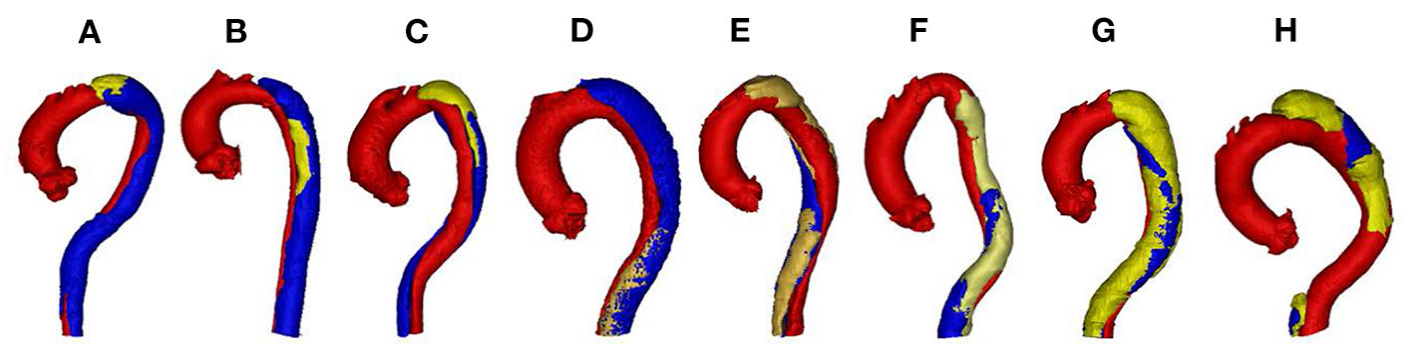
Examples of various relative position including **(A)** top, **(B)** middle, **(C)** top and middle, **(D)** bottom, **(E–G)** whole, and **(H)** multiple position in TBAD. The red, blue, and yellow parts correspond to TL, FL, and FLT, respectively. Best viewed in color.

**Figure 3:**
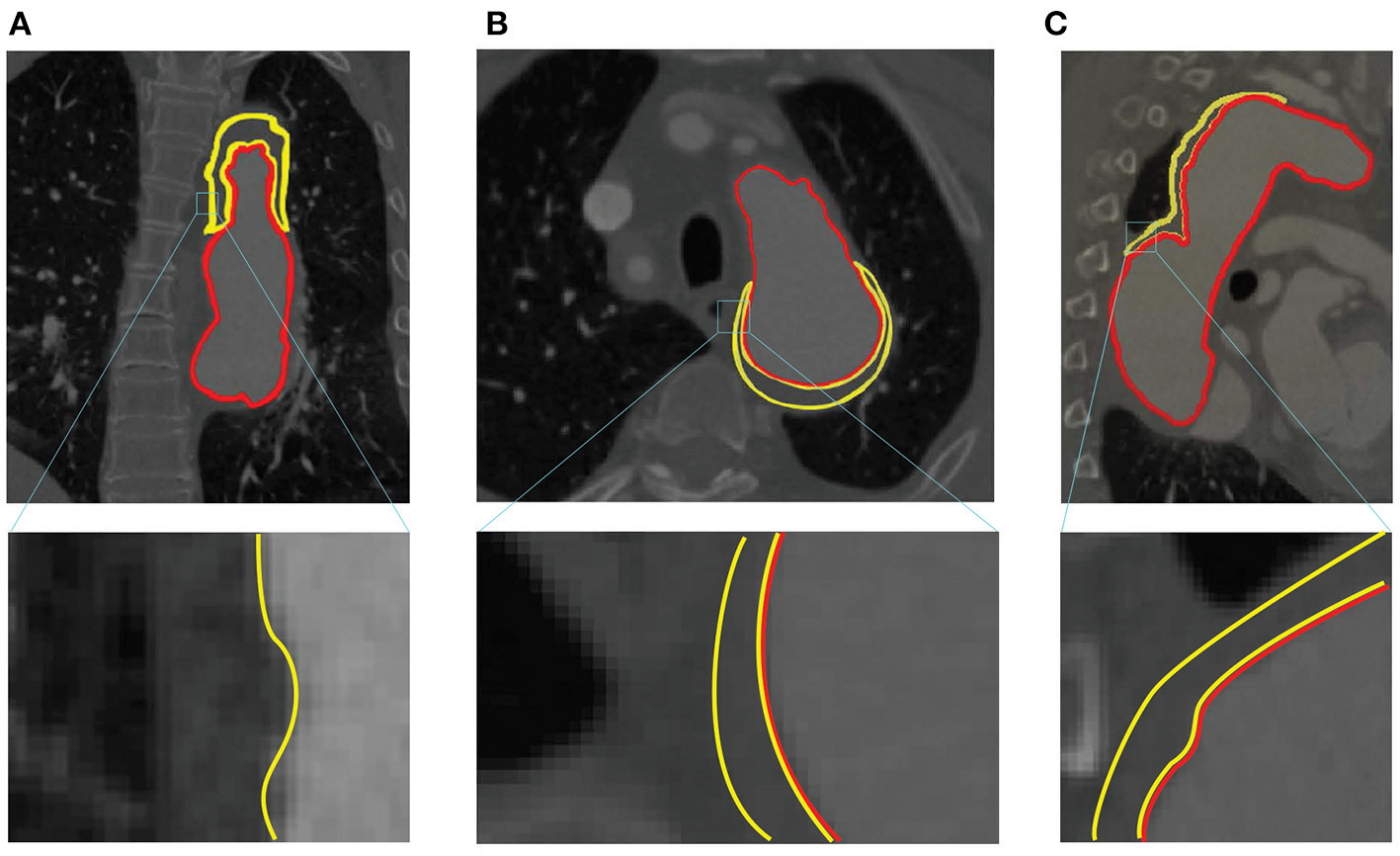
Example of low contrast images in the ImageTBAD dataset in three views: **(A)** coronal view, **(B)** axial view, and **(C)** sagittal view. Red and yellow lines denote to the boundary of TL and FLT, respectively.

By analyzing all the labels, we find the segmentation of FLT is challenging due to the following two reasons. First, FLT can appear almost anywhere along the aorta, with irregular shapes, although most FLT appear at the top of the aorta. Figure 2 shows a variety of relative positions of FLT. Figures 2A–C show the most common locations of FLT, while Figure 2D is also common in clinic. Figures 2E–H show some typical cases where FLT is distributed along with the whole FL and discontinued in multiple locations. Most FLTs exist at the surface of the aorta, but there are also some located at the center of the aorta and between the FL and the TF. Within the eight cases in Figure 2, we can notice the largest variety of the shapes of FLT. Most FLTs are rather thin and long, while some others are a pile at the top of the aorta. In addition, some FLTs are small which is relatively difficult to segment as shown in Figure 2G. Second, the contrast between FLT and other tissues is rather low. As shown in Figure 3, the intensity of the FLT and the nearby tissues are almost the same which is not easy to be visually recognized. By zooming the area of the boundary in, we can notice some parts of the boundary as shown in Figures 3A,B, but some are still with high uncertainty as shown in Figure 3C. The low contrast would bring more challenges to FLT segmentation.

## 3. Method and Experiment

### 3.1. The Baseline Method

By analyzing the dataset, we discover the following three phenomenons. First, the segmentation area is usually rather long in the axial view, which needs to be considered in the design of the input size. Second, the target segmentation is rather small compared with the size of the input, and processing the whole image is not efficient. Third, in most conditions, the combination of TL, FL, and FLT has a similar shape of the aorta. In fact, the part corresponding to FLT is a part of the aorta in normal anatomy. We can also obtain FLT by removing TL and FL from the combination of the three. This approach is expected to be more effective than direct segmentation of FLT because the complexity of shapes and positions of FLT can be avoided. For simplicity of discussion, the combination of the three parts is donated as the aorta.

**Figure 4:**
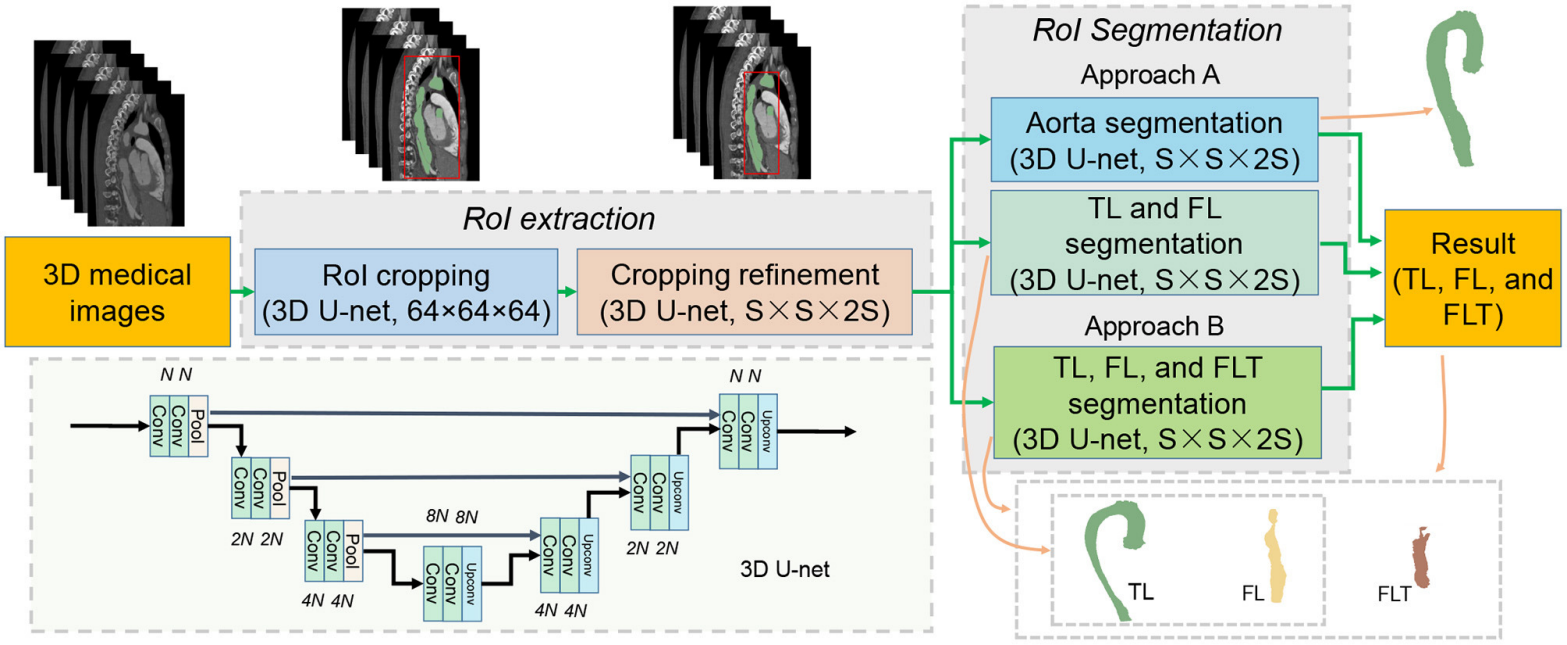
Processing pipeline of the proposed baseline method. Best viewed in color.

Based on the above observations, we propose a baseline method which is a processing pipeline shown in Figure 4. The processing pipeline includes two steps: region of interest (RoI) extraction, and RoI segmentation.

#### 3.1.1. RoI Extraction

The RoI extraction aims to obtain a precise bounding box of the target area, which is fulfilled with two croppings. The first cropping obtains a rough bounding box by segmenting the aorta on a resized input (original size to 64 × 64 × 64) using 3D U-net. Based on the rough bounding box, the rough RoI is cropped from the original input, and then resized to S × S × 2S. The cropping refinement is further proceeded on the rough RoI for aorta segmentation, and a relatively more precise bounding box of the RoI is obtained.

#### 3.1.2. RoI Segmentation

The RoI segmentation performs segmentation tasks on the refined RoI. We discuss two approaches: Approach A, we combine the TL and FL segmentation, and the aorta segmentation; Approach B, we perform direct segmentation of the three. In Approach A, we suppose to easily get FLT once we obtain both TL and FL and aorta according to our discovery. Note that all the modules adopt the same 3D U-net structure as shown in Figure 4. Four resolution levels are adopted each of which contains two convolutional layers and one pooling/up-convolutional layer. The number of filters is *N*, 2*N*, 4*N*, and 8*N* for the four resolution levels, respectively. *N* and the input size vary for different modules as discussed above. Post-processing only including upsampling to the original size is performed.

### 3.2. Experiment

#### 3.2.1. Experimental Setup

We implemented our baseline method using PyTorch based on Isensee et al. (2018). Both Dice loss and cross entropy loss were used, and the number of training epochs was 5 for all 3D U-nets. Data augmentation and normalization were also adopted with the same configuration as in Payer et al. (2017) for 3D U-net. For both networks and all the analyses, three-fold cross validation was performed (about 33 images for testing, and 67 images for training). We split the dataset so that the number of images containing FLT in each fold were the same. We implemented two configurations, with *S* = 64 and *S* = 96, respectively. Accordingly, *N* = 64 and the batch size was 4 when *S* = 64, and *N* = 32 and the batch size was 3 when *S* = 96. All the experiments ran on a Nvidia GTX 1080Ti GPU with 11 GB memory.

Dice score and Hausdorff distance were selected as the metrics for evaluation. For images without FLT, the Dice score is 1 if there is no FLT in the segmentation result, otherwise 0. As Approach B in RoI segmentation is similar to the methods that achieves the SOTA results in the TBAD (Li et al., 2018; Cao et al., 2019), we compared our method with theirs though their dataset and methods focused on the segmentation of FLT. Meanwhile, Hausdorff distance evaluated the shape similarity of propose method, which is formulated as follow,


(1) $$H\left(G,S\right)=\max\left\{\underset{x\in G}{\sup}\underset{y\in S}{\inf}||x-y||,\underset{y\in S}{\sup}\underset{x\in G}{\inf}||x-y||\right\},$$

where G and S represent ground truth and prediction segmentation, respectively.

#### 3.2.2. Statistical Analysis

Differences between results are compared using the independent two-sample *t*-test. A *p*-value of < 0.05 in the independent two-sample *t*-test is considered as statistical significance.

## 4. Results and Discussion

### 4.1. Overall Results

Tables 2, 3 demonstrate that the mean and standard deviation of Dice score and Hausdorff distance of baseline methods (Approach A and Approach B), and their *t*-test value and *p*-value for four substructures segmentation in TBAD, respectively. In terms of different substructures, both Approach A and Approach B achieves the highest scores on aorta with small Hausdorff distances. However, both two methods fail to segment the TL, FL, and FLT well, for the three are parts of the aorta without remarkable boundaries, thus relatively harder to segment them. The Dice score and Hausdorff distance of TL beats that of FL, which may be caused by the low contrast between FL and FLT. FLT obtains the lowest performance due to its great challenges discussed in section 2. As for the two methods, though Approach A with a multi-task segmentation module achieves a bit higher Dice score with a lower Hausdorff distance than Approach B using direct segmentation, it fail to achieve higher performance on the other two parts especially on FLT. Approach B obtains a large improvement over Approach A on FLT. This may due to the fact that direct segmentation has more constraint to more accurately define FLT than multi-task segmentation. On the other hand, we also notice some impacts from the input size. The Dice score of *S* = 96 is slightly higher than that of *S* = 64 due to the higher resolution of *S* = 96. However, the improvement is small, and there is no improvement for FLT, which indicates that higher resolution has very limited success on FLT segmentation. Particularly, for all the 32 images without FLT, the baseline method with both configurations correctly obtain the results with Dice score of 1, which indicates that the FLT segmentation accuracy for images with FLT are much lower (about 20%) than 52%.

**Table 2:** Mean and standard deviation of Dice score of baseline method, and *t*-test value between the Approach A and Approach B for four substructures segmentation in TBAD.

		**Approach A**	**Approach B**	***t*-value**	***p***
S = 64	TL	0.82 ± 0.08	0.79 ± 0.07	2.327	<0.05
	FL	0.72 ± 0.20	0.68 ± 0.20	1.166	<0.05
	FLT	0.44 ± 0.42	0.50 ± 0.40	0.853	<0.05
	Aorta	0.89 ± 0.03	–	–	–
S = 96	TL	0.86 ± 0.08	0.85 ± 0.07	0.776	<0.05
	FL	0.77 ± 0.22	0.78 ± 0.21	0.271	<0.05
	FLT	0.44 ± 0.43	0.52 ± 0.40	1.123	<0.05
	Aorta	0.91 ± 0.04	–	–	–

**Table 3:** Mean and standard deviation of Hausdorff distance of baseline method, and *t*-test value between the Approach A and Approach B for four substructures segmentation in TBAD.

		**Approach A**	**Approach B**	***t*-value**	***p***
S = 64	TL	298.4 ± 275.2	565.0 ± 222.4	6.213	<0.05
	FL	597.5 ± 1117.3	1089.5 ± 1161.8	2.517	<0.05
	FLT	1095.1 ± 1879.9	1641.7 ± 1591.8	1.829	<0.05
	Aorta	300.2 ± 273.6	–	–	–
S = 96	TL	516.3 ± 482.5	288.4 ± 426	30.334	<0.05
	FL	1273.1 ± 2554.4	643.7 ± 1999.5	1.599	<0.05
	FLT	1564.3 ± 3453.5	978.6 ± 2887.3	1.072	<0.05
	Aorta	667.4 ± 612.3	–	–	–

Existing works most relevant to ours are the works proposed by a group from Tsinghua University (Li et al., 2018; Cao et al., 2019) though the dataset and labels are different. The method Li et al. (2018) achieves Dice scores of 0.92, 0.85, and 0.85 on aorta, TL, and FL, respectively on the same machine (11 GB GPU memory) as ours. The improved version (Cao et al., 2019) obtains Dice scores of 0.93, 0.93, and 0.91, on aorta, TL, and FL, respectively on a more powerful machine (32 GB GPU memory). Compared with Li et al. (2018), ours achieves almost the same performance on aorta and TL, but much lower on FL. While compared with Cao et al. (2019), ours obtains comparable performance only on aorta, but much lower on TL and FL.

The comparable results on aorta indicates that our baseline method is also a powerful one. The gaps in TL and FL may due to the difference on datasets, labels, and method details. Though with these difference, we can still notice that accurate segmentation of FLT is rather challenging. We hope our dataset and baseline method could help fill the gap and tackle this challenge.

### 4.2. Good Segmentation

**Figure 5:**
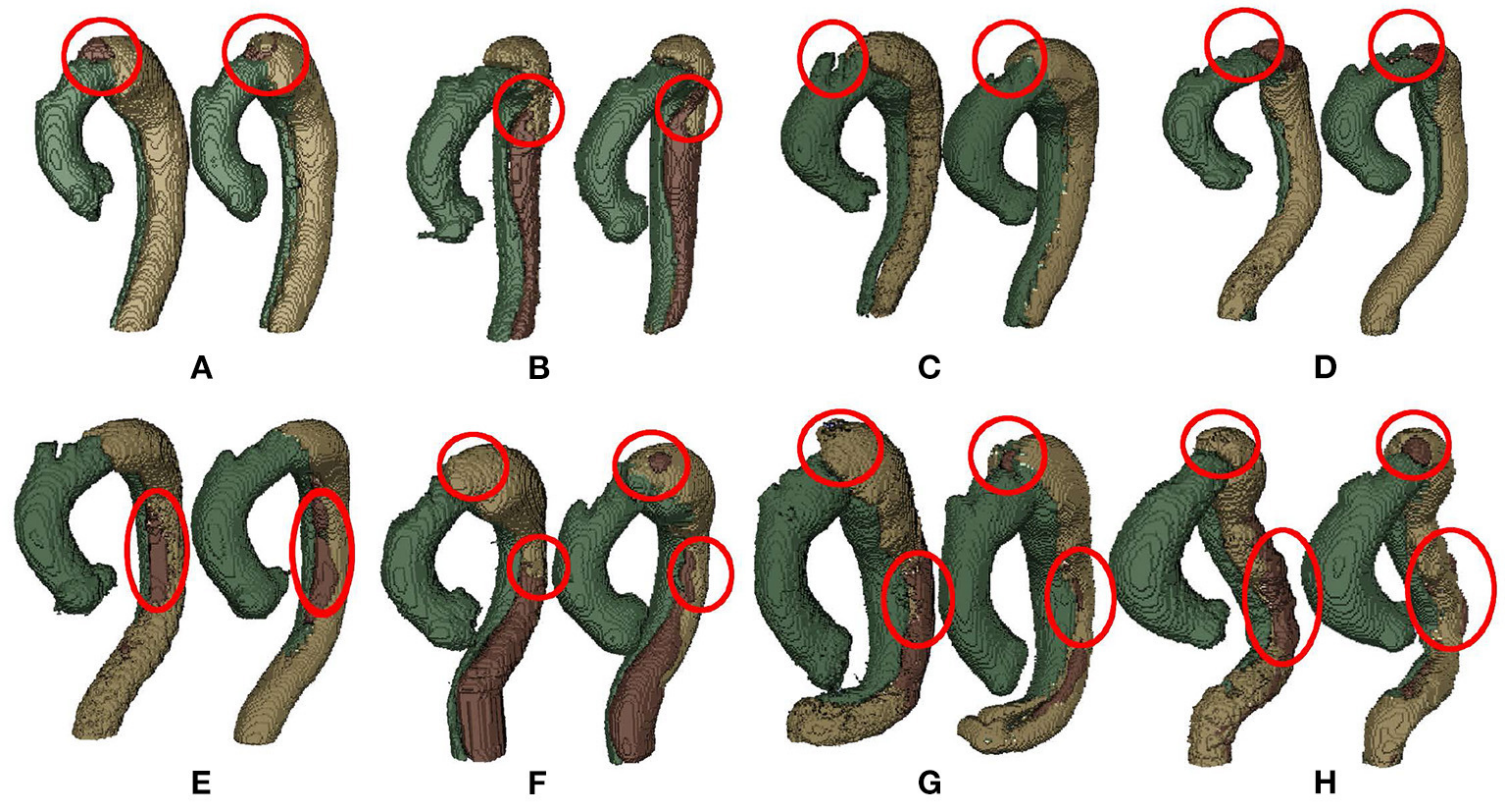
Examples of good segmentation results **(A,B,E,F)** with its corresponding ground truth **(C,D,G,H)**. Best viewed in color.

Examples of good segmentation results are shown in Figure 5. Overall, the segmentation results have a good match with the ground truth. However, we can still notice that compared with TL and FL, FLT has more segmentation flaws, which corresponds well to the Dice scores in Table 2. There is a tiny FL island at the top of the aorta which should be FLT as shown in Figure 5A. Another three tiny FLT islands exist at the similar position which should be FL as shown in Figures 5F–H, respectively. The most serious flaw of FLT is the inaccurate segmentation of its boundaries. As shown in Figures 5B,E,F, there is noticeable error of the boundary segmentation. The situations in Figures 5G,H is much worse, and a large part of FLT is misclassified as FL. Most of the inaccurate boundary segmentation happens at the descending aorta. Its low performance is usually caused by the low contrast, which also degrades the segmentation performance of FL. TL usually has a much better performance as its contrast is much higher, and there are only some tiny flaws as shown in Figure 5C.

### 4.3. Poor Segmentation

**Figure 6:**
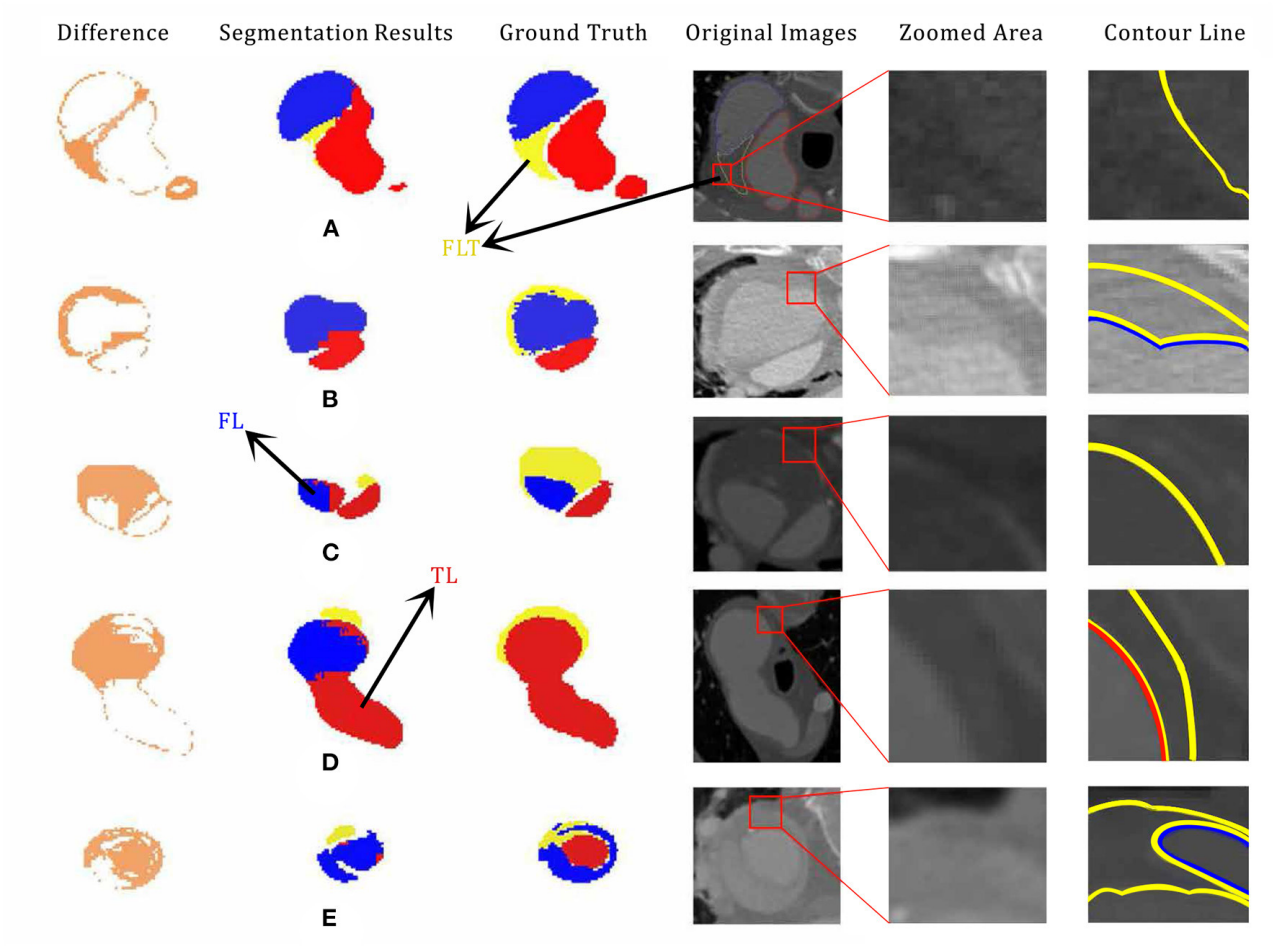
Examples of poor segmentation results **(A,B,C,D,E)** with their corresponding ground truth, segmentation difference, original CTA image, zoomed, and labeled CTA images. The original pictures and zoomed area of each cases are accompanied. segment failed part showed the impact of the shape and margin of thrombus in segmentation process. Especially, case **(D)** is the same CT scan picture of Figure 3B with 180 degrees flips vertically. Best viewed in color and position.

Examples of poor segmentation results are shown in Figure 6. Overall, there exists serious segmentation error especially for FLT. With the context of TL and FL, the shape of FLT in Figure 6A can be easily recognized by humans. However, only part of the shape is correctly segmented because of the low contrast as shown in the zoomed CTA image. A part of FLT is lost in Figures 6D,E which is due to the low contrast. The qualities get worse in both Figures 6B,C in which LFT are almost totally lost. The boundaries is difficult to visually tell in Figures 6B,C. There are also some inaccurate segmentation between TL and FL shown in Figures 6D,E. The incorrect connection exists between TL and FL in Figure 6D, and the low contrast in CTA images leads to the inaccurate segmentation between FL and TL as shown in Figure 6E.

## 5. Conclusion

In this paper we introduce the ImageTBAD dataset to the community, which is the first 3D computed tomography angiography (CTA) image dataset of TBAD with annotation of true lumen (TL), false lumen (FL), and false lumen thrombus (FLT). We further propose a baseline method based on 3D U-net for automatic segmentation of TBAD. Results show that the baseline method can achieve comparable results with existing works on aorta and TL segmentations. However, the segmentation accuracy of FLT is only 52%, which leaves large room for improvement and proves the challenge of our dataset. FLT segmentation represents a wide class of segmentation problems where targets exist in a variety of positions with irregular shapes. We hope that the open-sourced code of our baseline method and dataset can encourage the community to tackle this problem.

## Data Availability Statement

The raw data that supporting the findings of this study are available at https://github.com/XiaoweiXu/Dataset_Type-B-Aortic-Dissection, and from the corresponding author without any reservation, upon reasonable request.

## Ethics Statement

This work was approved by the Research Ethics Committee of Guangdong General Hospital, Guangdong Academy of Medical Science under Protocol No. 20140316. All procedures performed in studies involving human participants were in accordance with the ethical standards of the institutional and/or national research committee and with the 1964 Declaration of Helsinki and its later amendments or comparable ethical standards.

## Author Contributions

ZY, WX, HQ, and HY contributed to data collection. ZY, JZha, JZhu, YD, TW, and YS contributed to analysis and writing. MH, LQ, and XX contributed to project planning, development, discussion, and writing. All authors contributed to the article and approved the submitted version.

## Funding

This work was supported by the National key Research and Development Program of China (No. 2018YFC1002600), the Science and Technology Planning Project of Guangdong Province, China (Nos. 2017B090904034, 2017B030314109, 2018B090944002, 2019B020230003), Guangdong Peak Project (No. DFJH201802), the National Natural Science Foundation of China (No. 62006050).

## Conflict of Interest

The authors declare that the research was conducted in the absence of any commercial or financial relationships that could be construed as a potential conflict of interest.

## Publisher's Note

All claims expressed in this article are solely those of the authors and do not necessarily represent those of their affiliated organizations, or those of the publisher, the editors and the reviewers. Any product that may be evaluated in this article, or claim that may be made by its manufacturer, is not guaranteed or endorsed by the publisher.
